# Mammographic screening for breast cancer: A review

**DOI:** 10.1002/jmrs.6

**Published:** 2013-02-03

**Authors:** Warwick Lee, Gudrun Peters

**Affiliations:** 1 BreastScreen NSW, Cancer Institute NSWAlexandria, New South Wales, 1435, Australia; 2 Discipline Medical Radiation Sciences, The University of SydneyLidcombe, New South Wales, 2141, Australia; 3 Regional Imaging TasmaniaLenah Valley, Tasmania, 7008, Australia; 4 BreastScreen TasmaniaHobart, Tasmania, 7001, Australia

**Keywords:** Breast cancer, mammography, overdiagnosis, screening

## Abstract

In 2011, BreastScreen Australia celebrated 20 years of mammographic screening for breast cancer in Australia. There has been a reduction in mortality from breast cancer over the last two decades, coincident with mammographic screening. However, there are concerns that mammographic screening may result in overdiagnosis of breast cancer and that the reduction in mortality from breast cancer is the result of better treatment rather than screening. This article reviews the evidence on which mammographic screening for breast cancer is based, considers the issue of overdiagnosis of breast cancer by screening mammography, and assesses the role of screening mammography in the reduction in breast cancer mortality seen over the last two decades.

## Introduction

Breast cancer is the most common cancer in women, making up over 25% of all cancers in women, and is the second most common cause of cancer death in women after lung cancer.^1^ There has been a reduction in the mortality from breast cancer in the last 20 years, but 1 in 38 women still die from breast cancer,^1^ and it remains a major cause of illness in Australian women. In 2006, there were 12,614 women diagnosed with invasive breast cancer.^1^ This article reviews the evidence on which mammographic screening for breast cancer is based, describes the methods of mammographic screening used by BreastScreen Australia, and considers the mortality reduction from breast cancer due to mammographic screening and the issues of overdiagnosis and overtreatment that may be associated with mammographic screening for breast cancer.

## Randomized Trials

The first randomized controlled trial to show a mortality benefit of mammographic screening is known as the Health Insurance Plan of Greater New York or the HIP study. This study began in 1963 and was first reported in 1971.^2^ There have now been 10 randomized trials of mammographic screening with a combined reduction in mortality associated with mammographic screening of 21%.^3^ The Swedish 2 County trial was first reported in 1985,^4^ has now been followed up for 29 years,^5^ and reported a 27–31% reduction in mortality from breast cancer due to mammographic screening.

## BreastScreen Australia

The BreastScreen Australia Program was established based on the results of the randomized trials and on successful pilot screening programmes run in Australia from 1987 to 1990 by the National Breast Cancer Screening Evaluation.^6^ The program commenced in 1991 and was initially known as the National Program for the Early Detection of Breast Cancer.^6^ It has been known as BreastScreen Australia since 1996.

BreastScreen Australia is a population-based screening programme offering biennial mammography to a target population aiming to detect breast cancer at an early stage and to reduce mortality from breast cancer.^7^ The target population invited to have screening mammograms is composed of all women without symptoms of breast cancer aged 50–69 years. Women aged 40–49 and 70 years and older are eligible to be screened, but are not actively recruited by BreastScreen Australia. Currently, BreastScreen Australia operates in over 500 accredited locations nationwide.

The screening programme consists of two components: screening and assessment. Initially, two-view mammography is performed at screening sites. All screening mammograms are read independently by two radiologists (double reading). In approximately 90–95% of cases, women are reassured that there is no sign of cancer following the screening mammograms. In the remaining 5–10% of cases, an abnormality may be detected or suspected on the screening mammograms and the woman is invited or “recalled” to the assessment centre to confirm or exclude the presence of breast cancer. Of the women recalled to assessment, approximately 20% are found to have breast cancer. BreastScreen Australia assessment clinics utilize a multidisciplinary approach to allow comprehensive assessment of screen-detected abnormalities^8^ by clinical examination, further mammographic work-up, breast ultrasound, and percutaneous needle biopsy as appropriate. Members of the multidisciplinary team include radiologists, breast surgeons, radiographers, and nurse/counsellors.

## BreastScreen: Reduced Breast Cancer Mortality or Overdiagnosis

There is no doubt that both the survival from breast cancer and the mortality from breast cancer have improved over the last 20 years^1^,^9^ and that this coincides with the period of organized mammographic screening for breast cancer in Australia. The death rate from breast cancer in Australia has reduced from 30 deaths per 100,000 women in 1994 to a rate of 22 deaths per 100,000 women in 2006^1^ and survival at 5 years for all women in NSW with breast cancer has increased by 15% since 1982.^9^ However, improved survival after the diagnosis of breast cancer may be the result of earlier diagnosis (lead time bias) and diagnosis of cancers that may never have resulted in symptoms or death (overdiagnosis). Therefore, the efficacy of mammographic screening should be measured by its effect on breast cancer mortality, not survival.^10^

It has been estimated that BreastScreen Australia has been successful in reducing the death rate from breast cancer by 21–28%.^6^,^11^ A case–control study of BreastScreen South Australia demonstrated that participation in screening was associated with between 30% and 41% reduction in breast cancer mortality.^12^ A recent case–control study of participation in BreastScreen Western Australia (WA) demonstrated a 52% reduction in the mortality from breast cancer associated with participation in the WA BreastScreen programme.^13^ A recent review of all observational studies based on European population screening programmes has reported that case–control studies demonstrated a combined 48% reduction in mortality associated with participation in mammographic screening.^14^

In contrast, other studies in both Australia^15^ and internationally^16^–^18^ claim that there is little or no association between reduction in breast cancer mortality and mammographic screening. The conflicting results may be explained by study design.^13^,^14^ The studies that have shown reduction in mortality, such as the studies by Roder et al.^12^ and Nickson et al.,^13^ are case–control studies which compare prior screening activity of women who have died from breast cancer matched with controls who are still alive. The studies that have shown little or no benefit are generally ecological or trend studies which study trends in breast cancer mortality compared with mammographic screening activity at a population level, rather than at the individual level.^3^,^13^,^14^ For comparisons between screened and unscreened populations to be valid, ecological studies need to be able to accurately assess the levels of screening activity in different populations. This is a difficult task and leads to inconsistent results.^3^,^13^,^14^ In contrast to the period of randomized trials, there are virtually no control groups which are uncontaminated by opportunistic screening and heightened awareness of breast cancer.^10^ In addition, study design may lead to inaccurate results. A recent study^18^ reported that in contrast to the initially reported results of the Swedish 2 country trial^5^ which has consistently reported a 30% reduction in mortality for women invited to screening, this trial demonstrated no effect on mortality by mammographic screening. However, this study included women diagnosed with breast cancer before screening commenced and who died from breast cancer after screening commenced and such a study design can result in a 50% underestimation in the efficacy of mammographic screening.^19^

Studies have attempted to compare the proportional effect of screening and improved adjuvant therapy on breast cancer mortality.^15^,^20^ Such studies are reliant upon statistical modelling which may result in variable results.^10^ In one study,^20^ seven different groups used seven independent models to assess the same data. This study found that estimates of the proportional contribution to the reduction in breast cancer mortality from screening ranged from 28% to 65% with a median of 46%. An Australian study^15^ based on breast cancer mortality trends and BreastScreen Australia participation found that there was little, if any, contribution from mammographic screening. Such studies, based on trend data, may underestimate the efficacy of screening,^13^,^14^ but the study adds to the doubt and confusion regarding the efficacy of mammographic screening for breast cancer.

Overdiagnosis of cancer is the diagnosis of a cancer that would never result in symptoms during a person's lifetime or cause death.^21^ Such cancers may be slowly growing cancers or indolent cancers that do not progress. Overdiagnosis may be equivalent to overtreatment and represents a risk of screening. There has been an increase in the incidence of breast cancer in Australia over the last 50 years.^1^ Some of the increased incidence is related to life-style issues such as the use of hormone replacement therapy and obesity, but the most dramatic increase in incidence coincided with the commencement of the BreastScreen Australia Program.^1^ Estimates of the rate of overdiagnosis vary and are dependent on models used to estimate overdiagnosis.^22^ It has been estimated that in NSW, there is a 30% overdiagnosis rate after adjustments for underlying breast cancer risk and lead time bias,^23^ a rate similar to that predicted by analysis of other screening programmes.^24^ In contrast, a recent literature review of overdiagnosis in European screening programmes found that the overdiagnosis rate varied between 1% and 10% after adjustment for the underlying breast cancer risk and lead time bias.^22^

## Informed Consent

Women who are invited to participate in BreastScreen are asked to provide informed consent for screening. Historically, they have been provided with information regarding the benefits of screening with regard to improved survival and reduction in breast cancer mortality. Programmes have informed women that screening mammography does not detect all cancers. However, overdiagnosis has not generally been explained to women invited to screening, and there have been calls to ensure that women are able to provide fully informed consent.^25^ This need is well recognized by those involved with organized screening programmes such as BreastScreen Australia.^26^ The consent form for BreastScreen Victoria has recently been changed to include a section on overdiagnosis, and the website of BreastScreen NSW^27^ has just been redeveloped and now includes a section on overdiagnosis. Cancer Australia has a position statement on overdiagnosis,^28^ initially produced in 2008, revised in 2010, and currently under further revision, due for release in October 2012.

It is important to inform women invited to mammographic screening about overdiagnosis. They should be informed of its existence, but just as importantly, they should be informed that at present, it is not possible to determine which cancers are overdiagnosed by mammographic screening. There are clinical tools that predict risk and prognosis which help clinicians and patients make informed decisions about treatment and attempt to avoid overtreatment. Genetic profiling of tumours may allow identification of low-risk tumours,^29^ but such molecular profiling of tumours is not widely available and the utility of molecular profiling is not yet proven.

At present, mammographic screening is a balance between the benefit of reduction in mortality and the risk of overdiagnosis and possible overtreatment. It is estimated that between 2 and 2.5 lives are saved for every case of overdiagnosis.^30^,^31^ The use of a balance sheet that clearly presents the benefits and risks to women has been proposed by the EUROSCREEN working group^32^ for organized screening programmes in Europe.^31^ Such balance sheets could be provided to women invited to screening to allow informed consent to be made.

## Alternative Screening Modalities

While screening mammography is the only imaging modality proven to decrease breast cancer mortality in the general population,^3^ breast ultrasound, breast MRI, and more recently, breast tomosynthesis have roles or possible roles in screening for breast cancer.

### Breast ultrasound

Breast ultrasound has a well-established role as a targeted examination following an abnormal clinical examination or abnormal screening mammogram. Screening whole breast ultrasound has been shown to increase the detection rate of breast cancer by up to 55% in women with dense breasts when added to screening mammography.^33^ However, this increase in sensitivity comes at the cost of reduced specificity and reduced positive predictive value of needle biopsy following an abnormal ultrasound.^33^ There is insufficient evidence of the efficacy of screening breast ultrasound, even in women with dense breasts, to be able to recommend its routine use.

### Magnetic resonance imaging

Contrast-enhanced breast MRI is the most sensitive imaging modality for the detection of breast cancer.^34^ It has a role in screening for breast cancer in women with a high risk of breast cancer,^35^–^37^ but it is not used for screening for breast cancer in the general population, primarily due to its expense and limited access, compared with screening mammography. Women considered at high risk are those with a strong family history, such as women with multiple first-degree relatives diagnosed with breast cancer at <50 years of age,^38^ and women who are carriers of genetic mutations in the *BRCA1* and *BRCA2* genes or their relatives.^38^

### Tomosynthesis

Breast tomosynthesis enables the reader to view mammographic images as a series of thin reconstructed sections through the breast. A digital mammogram records a series of low-dose images while traversing a small arc around the compressed breast. With every projection, the angle changes and obtained images differ in depths and thickness through the breast. This technique can reduce the superimposition of normal breast tissue seen in two-dimensional mammography. Preliminary results of breast tomosynthesis show that it could improve the accuracy of breast cancer detection.^39^,^40^ Uncertainty remains as to whether breast tomosynthesis can be used in screening and assessment settings and further studies are required.

## Conclusion

There is a highly significant reduction in breast cancer mortality associated with participation by women in the BreastScreen Australia Program.^12^,^13^ Overdiagnosis needs to be considered and women invited to screening must be informed of the risks and benefits of mammographic screening. They also need to be informed that at present, cancers that are overdiagnosed cannot be distinguished from potentially lethal cancer. It is estimated that 2–2.5 lives are saved for each case of overdiagnosis.^30^,^31^

## References

[1] Breast cancer in Australia: An overview (2009). http://www.aihw.gov.au/publication-detail/?id=6442468297&tab=2.

[2] Shapiro S, Strax P, Venet L (1971). Periodic breast cancer screening in reducing mortality from breast cancer. JAMA.

[3] Smith R, Duffy S, Tabar L (2012). Breast cancer screening: The evolving evidence. Oncology.

[4] Tabar L, Fagerberg CJ, Gad A (1985). Reduction in mortality from breast cancer after mass screening with mammography: Randomised trial from Breast Cancer Screening Working Group of the Swedish National Board of Welfare. Lancet.

[5] Tabar L, Vitak B, Chen T (2011). Swedish-two county trial: Impact of mammographic screening on breast cancer mortality during 3 decades. Radiology.

[6] BreastScreen Evaluation http://www.cancerscreening.gov.au/internet/screening/publishing.nsf/Content/br-evaluation-report-cnt.

[7] http://www.cancerscreening.gov.au/internet/screening/publishing.nsf/Content/national-policy.

[8] http://www.cancerscreening.gov.au/internet/screening/publishing.nsf/Content/br-accreditation/$File/standards.pdf.

[9] Cancer Survival in NSW 2002–2006 http://www.cancerinstitute.org.au/media/177460/cancer-survival-in-nsw-2002-06.pdf.

[10] Bulliard J-L, Levi F (2012). Mammography screening: Time to reevaluate its impact?. Eu J Cancer Prev.

[11] Morrell S, Taylor R, Roder D, Dobson A (2012). Mammography screening and breast cancer mortality in Australia: An aggregate cohort study. J Med Screen.

[12] Roder D, Houssami N, Farshid G, Gill G, Luke C, Downey P, Beckmann K, Iosifidis P, Grieve L, Williamson L (2008). Population screening and intensity of screening are associated with reduced breast cancer mortality: Evidence of efficacy of mammography screening in Australia. Breast Cancer Res Treat.

[13] Nickson C, Mason KE, English DR, Kavanagh AM (2012). Mammographic screening and breast cancer mortality: A case-control study and meta-analysis. Cancer Epidemiol Biomarkers Prev.

[14] Broeders M, Moss S, Nystrom L, Njor S, Jonsson H, Paap E, Massat N, Duffy S, Lynge E, Paci E (2012). The impact of mammographic screening on breast cancer mortality in Europe: A review of observational studies. J Med Screen.

[15] Burton R, Bell R, Thiagarajah G, Stevenson C (2012). Adjuvent therapy, not mammographic screening, accounts for most of the observed breast cancer specific mortality reductions in Australian women since the national screening program began in 1991. Breast Cancer Res Treat.

[16] Jorgensen K, Zahl P-H, Gotzsche P (2010). Breast cancer mortality in organised mammography screening in Denmark: Comparative study. BMJ.

[17] Autier P, Bonilo M, Gavin A, Vatten L (2011). Breast cancer mortality in neighbouring European countries with different levels of screening but similar access to treatment: Trend analysis of WHO mortality data base. BMJ.

[18] Autier P, Koechlin A, Smans M, Vatten L, Boniol M (2012). Mammography screening and breast cancer mortality in Sweden. J Natl Cancer Inst.

[19] Tabar L http://www.auntminnie.com/index.aspx?sec=sup&sub=wom&pag=dis&itemID=99977.

[20] Berry D, Cronin K, Plevritis S, Fryback D, Clarke L, Zelen M, Madelblatt JS, Yakovlev AY, Habbema DF, Feuer EJ (2005). Effect of screening and adjuvant therapy on mortality from breast cancer. N Engl J Med.

[21] Welch H, Black W (2010). Overdiagnosis in cancer. J Natl Cancer Inst.

[22] Puliti D, Duffy S, Miccenesi G, de Konig H, Lynge E, Zappa M (2012). Overdiagnosis in mammography screening for breast cancer in Europe: A literature review. J Med Screen.

[23] Morrell S, Barratt A, Irwig L, Howard K, Biesheuvel C, Armstrong B (2010). Estimates of overdiagnosis of invasive breast cancer associated with screening mammography. Cancer Causes Control.

[24] Jorgensen J, Gotzsche P (2009). Overdiagnosis in publicly organised screening programs: Systematic review of incidence trends. BMJ.

[25] Barratt A, Glasziou P (2012). Do the benefits of screening mammography outweigh the harms of overdiagnosis and unnecessary treatment?. Med J Aust.

[26] Roder D, Olver I (2012). Do the benefits of screening mammography outweigh the harms of overdiagnosis and unnecessary treatment? – Yes [opposing views]. Med J Aust.

[27] BreastScreen NSW http://www.bsnsw.org.au/about-mammograms/benefits-and-limitations.

[28] Cancer Australia http://canceraustralia.nbocc.org.au/our-organisation/position-statements/over-diagnosis-from-mammography-screening.

[29] Esserman L, Shieh Y, Rutgers E (2011). Impact of mammographic screening on the detection of good and poor prognosis breast cancers. Breast Cancer Res Treat.

[30] Duffy SW, Tabar L, Olsen AH, Vitak B, Allgood PC, Chen TH, Yen AMF, Smith RA (2010). Absolute numbers of lives saved and overdiagnosis in breast cancer screening, from a randomized trial and from the Breast Cancer Screening Programme in England. J Med Screen.

[31] EUROSCREEN Working Group (2012). Summary of the evidence of breast cancer service screening outcomes in Europe and first estimate of benefit and harm balance sheet. J Med Screen.

[32] Hackshaw A (2012). The benefits and harms of mammographic screening for breast cancer: Building the evidence base using service screening programmes. J Med Screen.

[33] Berg WA, Blume JD, Cormack JB (2008). Combined screening with ultrasound and mammography vs. mammography alone in women at elevated risk of breast cancer. JAMA.

[34] Linda A, Zuiani C, Londero V, Bazzocchi M (2008). Outcome of initially only magnetic resonance mammography – Detected findings with and without correlate at second-look sonography: Distribution according to patient history of breast cancer and lesion size. Breast.

[35] Kuhl CK, Schrading S, Leutner CC, Morakkabati-Spitz N, Wardelmann E, Fimmers R, Kuhn W, Schild HH (2005). Mammography, breast ultrasound and magnetic resonance imaging for surveillance of woman at high familial risk for breast cancer. J Clin Oncol.

[36] Lord SJ, Lei W, Craft P, Cawson JN, Morris I, Walleser A, Griffiths A, Parker S, Houssami N (2007). A systematic review of the effectiveness of magnetic resonance imaging (MRI) as an addition to mammography and ultrasound in screening young women at high risk of breast cancer. Eur J Cancer.

[37] Taylor D, Wylie E http://www.insideradiology.com.au/pages/view.php?T_id=19&ref_info.

[38] http://canceraustralia.gov.au/clinical-best-practice/gynaecological-cancers/familial-risk-assessment-fra-boc.

[39] Michell MJ, Iqbal A, Wasan RK, Evans DR, Peacock C, Lawinski CP, Douiri A, Wilson R, Whelehan P (2012). A comparison of the accuracy of film-screen mammography, full-filed digital mammography and digital breast tomosynthesis. Clin Radiol.

[40] Svahn TM, Chakraborty DP, Ikeda D, Zackrisson S, Mattsson S, Andersson (2012). Breast tomosynthesis and digital mammography: A comparison of diagnostic accuracy. Br J Radiol.

